# A comparison of the folding characteristics of free and ribosome-tethered polypeptide chains using limited proteolysis and mass spectrometry

**DOI:** 10.1002/pro.2702

**Published:** 2015-06-11

**Authors:** Khadijeh Rajabi, Julia Reuther, Elke Deuerling, Sheena E Radford, Alison E Ashcroft

**Affiliations:** 1 Astbury Centre for Structural Molecular Biology, School of Molecular and Cellular Biology, University of LeedsLeeds LS2 9JT, United Kingdom; 2 Department of Biology, University of Konstanz78457, Konstanz, Germany

**Keywords:** ribosome-nascent chain complexes, limited proteolysis, nanoelectrospray ionisation-mass spectrometry, circular dichroism spectroscopy, Src-homology 3, trypsin digest

## Abstract

The kinetics and thermodynamics of protein folding are commonly studied *in vitro* by denaturing/renaturing intact protein sequences. How these folding mechanisms relate to *de novo* folding that occurs as the nascent polypeptide emerges from the ribosome is much less well understood. Here, we have employed limited proteolysis followed by mass spectrometry analyses to compare directly free and ribosome-tethered polypeptide chains of the Src-homology 3 (SH3) domain and its unfolded variant, SH3-m10. The disordered variant was found to undergo faster proteolysis than SH3. Furthermore, the trypsin cleavage patterns observed show minor, but significant, differences for the free and ribosome-bound nascent chains, with significantly fewer tryptic peptides detected in the presence of ribosome. The results highlight the utility of limited proteolysis coupled with mass spectrometry for the structural analysis of these complex systems, and pave the way for detailed future analyses by combining this technique with chemical labeling methods (for example, hydrogen-deuterium exchange, photochemical oxidation) to analyze protein folding in real time, including in the presence of additional ribosome-associated factors.

## Introduction

Protein folding mechanisms have been studied extensively on purified proteins in the >40 years since Anfinsen's initial studies.^1^ These experiments have revealed details of folding energy landscapes and of how sequences determine the complexity of folding to a native state.^2^ However, the way in which a nascent polypeptide initiates folding as it emerges from the ribosome exit tunnel is much less well understood. Indeed, the ribosome is known to interact with nascent chains during translation, and folding of the nascent chain is assisted by chaperones immediately upon its emergence from the ribosome exit tunnel.^3^,^4^ The surface of the ribosome may also alter the kinetics and/or thermodynamics of the folding process compared with *in vitro* refolding studies. For example, the chaperoning abilities of the *E. coli* ribosome (70S) have been shown to prevent aggregation of partially folded protein intermediates.^5^ The ribosome has also been found to retard folding of two-domain proteins via decelerating the formation of stable tertiary interactions and the attainment of the native state.^6^ There is a high degree of functional and structural conservation between bacterial and eukaryotic ribosomes and conserved rRNA elements of the large ribosomal subunit are assumed to have chaperoning functions. Accordingly, the protein folding ability is also observed for eukaryotic ribosomes, e.g. from yeast, rat liver or wheat germ, and seems to be a universal property of the translation machinery.^5^

The synthesis of proteins by ribosomes *in vivo* proceeds in a vectorial fashion commencing from the N-terminus. Thus, the folding of nascent polypeptides may be different from the refolding of full-length denatured proteins wherein the full sequence is available to form interactions throughout the folding process. Sequential folding of domains during synthesis on the ribosome depends on the nascent polypeptide. In most small single-domain proteins, complete emergence of the nascent polypeptide is required before co-translational folding occurs.^7^ Alternatively, some proteins start to attain secondary and tertiary structures immediately after they begin to emerge from the ribosomal exit tunnel.^8^,^9^ Domain-wise folding is proposed to be the efficient folding pathway for many eukaryotic multi-domain proteins.^10^ The ribosomal exit tunnel, on average 20 Å wide, can potentially accommodate an α-helix. In addition to being a route from which to liberate the nascent chain, the exit tunnel wall has been found to form specific interactions with the nascent polypeptide.^11^,^12^ Indeed, nascent chain-tunnel interactions are used for drug sensing by the ribosome^13^ and some small molecules can be employed to stall ribosomes via interaction with the exit tunnel.^14^,^15^

**Figure 1:**
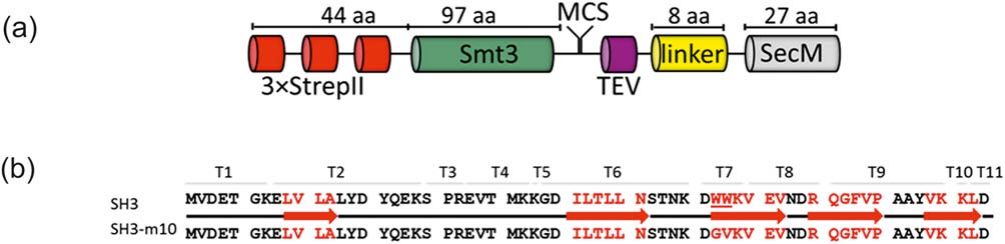
The sequence and display of Src-homology 3 (SH3) domains on stalled ribosomes. (a) Plasmid for expressing nascent chains on stalled ribosomes.^7^ The nascent chain contains an N-terminal triple StrepII tag (red) followed by a Smt3 domain (green), a multiple cloning site (MCS), a recognition sequence for the TEV protease (magenta), an eight amino acid residue linker (yellow), and the 27 amino acid residue SecM stalling peptide (gray); (b) the amino acid sequence and the secondary structure of SH3 and SH3-m10. Peptides resulting from tryptic digestion are indicated above the sequence; for example, T1 represents resides 1–7 following cleavage at the first basic site, K7. Red arrows indicate the location of the β-strands within the protein sequence according to the crystal structure of SH3.^26^

Here we have generated stalled *E. coli* ribosomes^7^ using a 27-residue peptide, SecM,^16^ with the motif ^150^FxxxxWIxxxxGIRAGP^166^ which interacts strongly with the ribosome and causes translation to stop. The construct used to generate ribosome-nascent chain complexes (RNCs) is shown in Figure 1(a). The SecM sequence is genetically fused via an 8-residue linker (GASGGASG) and a recognition site for TEV protease (ENLYFQG) to the C-terminal end of the nascent polypeptide. This assures the exposure of the nascent polypeptide at the outside of the ribosomal exit tunnel. The multiple cloning site (MCS) enables introduction of a target gene encoding a nascent polypeptide of choice into the vector. The N-terminus of the nascent polypeptide is attached to a triple StrepII-tag with a small ubiquitin-related modifier (Smt3)-domain in between. The 8-residue StrepII tag (WSHPQFEK) enables affinity purification of the stalled RNCs by forming a complex with streptavidin.^17^ Smt3 is recognized by the Ulp1 protease (Ubiquitin-like-specific protease 1) that cleaves the polypeptide downstream of Smt3, producing nascent chains with a correct (i.e. native) N-terminus.^18^

We have used limited proteolysis followed by mass spectrometric analysis to compare directly free and ribosome-tethered polypeptide chains of the Src-homology (SH3) domain and its unfolded variant, SH3-m10. To achieve this, we have generated two types of RNCs: SH3-RNC and SH3-m10 RNC, together with their corresponding intact sequences (i.e., isolated polypeptides).^7^ The SH3 domain from α-spectrin (63 residues, Δ*G*_F-U_ = 3.9 kcal mol^−1^) consists of five β-strands and was used as a model for a folded chain [Fig. 1(b)]. SH3-m10, the W42G, and W43V variant of SH3, is unable to fold and served as a model for an unfolded state.^19^ Thus, the structure and stability of the polypeptide sequence could be compared both in its native and unfolded forms and in the context of display at the ribosome exit site.^7^ We then used limited proteolysis of both the free and ribosome-bound nascent chains followed by nanoelectrospray ionisation-mass spectrometry (nESI-MS) to compare directly the structure and dynamics of the ribosome-tethered polypeptide chains with their free counterparts. The data show that the trypsin cleavage rate is different for the free and ribosome-bound nascent chains, with less extensive digestion detected in the presence of the ribosome. Moreover, using nESI-MS, peptides released from the nascent chains could be distinguished from a wealth of peptides originating from the ribosomes themselves. The results highlight the power of MS-based approaches, in terms of speed, sensitivity, and the ability to characterize individual components within heterogeneous samples, for the analysis of RNCs. They also pave the way for further detailed studies, for example using chemical labelling techniques such as hydrogen-deuterium exchange, photochemical oxidation, and cross-linking combined with sequence analysis using tandem MS.

## Experimental Section

### Design and purification of ribosome-nascent chain complexes

The plasmid [Fig. 1(a)] was used to generate RNCs as described previously.^7^ Free proteins were expressed recombinantly and purified as described in Ref. 19. Note that the proteins contain an additional N-terminal methionine (numbered M1) compared with the wild-type sequence.

### Circular dichroism spectroscopy

Far-UV circular dichroism (CD) spectra for all isolated protein variants (8 µ*M* in 10 m*M* ammonium acetate buffer at pH 6.0) were recorded on a Chirascan spectrophotometer (Applied Photophysics, Leatherhead, Surrey, UK), equipped with a Peltier temperature controller, in a 1.0 mm cuvette, with 4 nm bandwidth. The secondary structure content was estimated from the far-UV CD spectrum in the 180−260 nm range of each protein variant using Applied Photophysics Pro-Data and Global 3 Analysis software. Protein stability was analysed by monitoring the CD signals at 210, 224, and 228 nm upon thermal denaturation. Thermal runs were performed from 20 to 90°C at a rate of 1°C min^−1^. After thermal denaturation, protein samples were cooled to the starting temperature to confirm the reversibility of denaturation.

### Limited proteolysis

**Figure 2:**
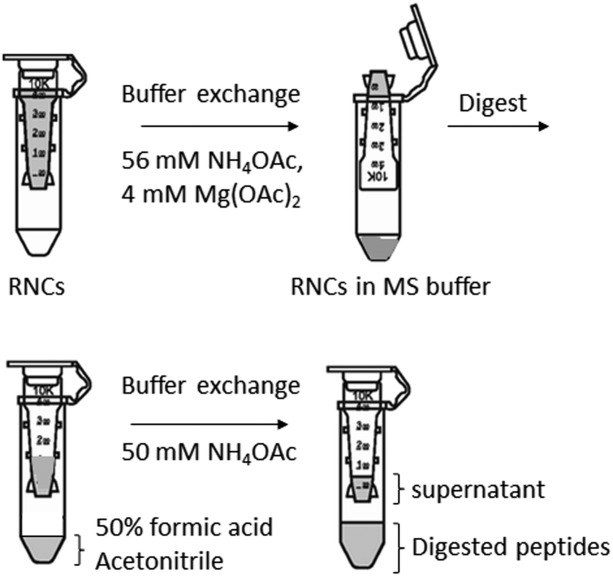
Experimental procedure for the limited proteolysis of RNCs. RNCs were buffer exchanged into 56 m*M* ammonium acetate, 4 m*M* magnesium acetate at 4°C and then subjected to limited proteolysis with trypsin (pH 6.8, 20°C). The resulting peptide fragments were separated from intact RNCs in the filtrate collection tube of a second filter and buffer exchanged into 50 m*M* ammonium acetate. To fix the digestion time at 10 min, the filtrate collection tube was supplied with 10 µL of 50% formic acid, 20 µL of acetonitrile prior to collecting the digested peptides. See also Experimental Section.

RNCs were buffer exchanged into 56 m*M* ammonium acetate, 4 m*M* magnesium acetate at 4°C using Amicon Ultra-0.5 YM-30 centrifugal filter devices (Merck Millipore, Darmstadt, Germany) and then subjected to limited proteolysis with trypsin (quantities as stated in the Results section) for 10 min at 20°C, pH 6.8. The peptides were separated from intact RNCs in the filtrate collection tube of a second filter with 30 MWCO and buffer exchanged into 50 m*M* ammonium acetate pH 6.8. To quench any further possibility of digestion, 10 µL of 50% (v/v) aqueous formic acid and 20 µL of acetonitrile were added to the collection vial prior to MS analysis (Fig. 2). Stoichiometric equivalents of each isolated protein were digested under the same conditions as RNCs, that is, trypsin at 20°C, pH 6.8, 10 min (Fig. 2).

### nESI-MS, nESI-MS/MS, and nESI-IMS-MS

Following proteolysis of isolated and ribosome-bound SH3, SH3-m10, the tryptic digest mixtures were introduced into a quadrupole-traveling wave ion mobility spectrometry (TWIMS)-time-of-flight mass spectrometer (Synapt HDMS, Waters Corpn., Manchester, UK)^20^ by Z-spray nanoESI (nESI). The mass spectrometer was operated in positive ion mode with in-house fabricated gold/palladium coated nESI capillaries at 1.5 kV, a source temperature of 40°C, and a backing pressure of 1.6 mbar. The sampling and extraction cone voltages were 40 V and 3 V, respectively. Ions were extracted into the flight tube (1 × 10^−6^ mbar) and detected by a dual microchannel plate at 1750 V. Mass calibration was performed with a CsI solution (1 mg mL^−1^ in 30% aqueous MeOH).

Solutions of peptides resulting from tryptic digests were sequenced using conventional collision induced dissociation (CID) in the trap region of the Tri-wave cell of the Synapt HDMS. Precursor ions were *m/z* selected in the quadrupole and fragmented in the trap cell with an optimized collision energy value in the 20−50 V range. The trap/transfer pressure was maintained at 8 × 10^−3^ mbar with an argon flow of 1.2 mL min^−1^.

The MS data were analyzed using MassLynx V4.1 and BioLynx software (Waters Corpn., Manchester, UK).

To measure rotationally averaged collision cross-sectional (CCS) areas, the mass spectrometer was operated in the ion mobility mode with a traveling wave height of 6 V, a wave velocity of 300 m s^−1^, and a nitrogen bath gas flow rate of 20 mL min^−1^. The injection voltages into the trap and transfer cells, located before and after the TWIMS cell, were 5 and 2 V, respectively. The CCS values were obtained via calibration with native-like protein standard calibrants.^21^ Data were processed using DriftScope software (Waters Corpn., Manchester, UK). The theoretical CCS of the native protein was estimated from the X-ray coordinates of the protein (PDB code 1SHG) as described previously.^22^

## Results and Discussion

### Characterization of isolated SH3 and SH3-m10

To compare the conformational properties of free and ribosome-tethered SH3 and SH3-m10 polypeptide chains, the conformational properties of these proteins in the absence of ribosome were first examined using CD, nESI-MS, and limited proteolysis.

### Far-UV CD

**Figure 3:**
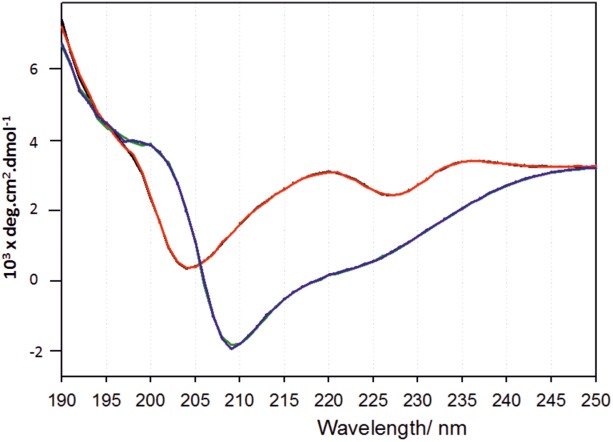
Far UV-CD characterization of isolated SH3 (red) and SH3-m10 (blue) acquired at a protein concentration of 8 µ*M* in 10 m*M* ammonium acetate, pH 6.0.

Far-UV CD spectra of SH3 and SH3-m10 in MS-compatible buffers are shown in Figure 3. For SH3, the spectrum has two negative maxima at 205 and 228 nm, characteristic of native SH3.^23^ Consistent with this, the protein denatures co-operatively with a melting temperature mid-point of 63.5 ± 1°C (data not shown). By contrast, the CD spectrum of SH3-m10 has a dominant negative band around 210 nm, indicative of an unfolded state (Fig. 3). Thermal denaturation of SH3-m10 indicates an absence of co-operative unfolding for this species (data not shown).

### nESI-MS and nESI-IMS-MS

The conformational properties of SH3 and SH3-m10 were next studied using nESI-MS to characterize the isolated polypeptides further. Analysis from 10 m*M* ammonium acetate solution at pH 5.8 generated a charge state distribution which highlighted the structural differences between the native and unfolded polypeptides (Supporting Information Fig. S1). For native SH3, only three charge states (+3 to +5) were detected (Supporting Information Fig. S1b), consistent with the charge-to-mass correlation expected for a folded, globular protein.^24^ By contrast, SH3-m10 has a wider charge state distribution (from +3 to +8) under the same solution conditions (Supporting Information Fig. S1d), indicative of a more extended and dynamic conformeric fingerprint. On decreasing the pH to 3.2, changes are observed in the ESI mass spectrum of SH3-m10: the +6 to +8 charge states ions are more highly populated and two additional, more highly charged species (+9 and +10 ions) appear. In the case of SH3, which populates a compact conformer at pH 5.8, the pH had to be lowered to pH 2 to denature the protein as apparent from the widening of the charge state distribution (+4 to +10 ions), similar to the distribution observed with SH3-m10 at pH 3.2, with the bimodal charge state distribution showing approximately equal intensity for the two populations (Supporting Information Fig. S1a,c). As both SH3 and SH3-m10 have equal numbers of basic amino acid residues (eight lysine residues and two arginine residues each), in addition to the N-terminus,^25^ the data suggest that under denaturing conditions in acidic pH solution these residues are exposed to solvent and are protonated to approximately the same extent. However, under nondenaturing solution conditions, the mass spectra indicate SH3 to be in a compact, folded state, whereas SH3-m10 is significantly more unfolded and dynamic.

Complementary data from nESI-IMS-MS analyses measuring the collision cross-sectional (CCS) area of the proteins together with their *m/z* in a single experiment corroborated the conformational differences between SH3 and SH3-m10 (Supporting Information Fig. S2). The +4 charge state ions of SH3 indicated the presence of two species: a more compact conformer of CCS 933 Å^2^ and a more extended (by ∼14%) conformer of 1071 Å^2^. A single conformer of CCS 1054 Å^2^ was observed for the corresponding +4 ions of SH3-m10, indicating that SH3 populates, at least to some extent, a slightly more compact conformeric species for the lowest charge state measured. Two conformers were observed in the case of the +5 charge state ions for both SH3 and SH3-m10; the CCS values, 1233/1412 Å^2^ and 1206/1380 Å^2^, respectively, were similar (∼2 %) and within experimental error. However, the +6 charge state ions of SH3-m10 populated a single conformer (1527 Å^2^) whereas the corresponding SH3 +6 ions populated three conformers (1213, 1385, and 1538 Å^2^), the most extended of which is of similar CCS to the SH3-m10 conformer, whilst the other two are significantly more compact. The more extended conformers observed for SH3-m10 populated +7 and +8 charge states with CCS values of 1515/1658 and 1793 Å^2^, respectively, were all absent in the case of SH3. Whilst SH3 and SH3-m10 populate some conformers of similar CCS for the +4 to +6 charge states (∼1000 and ∼1200 Å^2^), the +4 and +6 ions of SH3 also populate more compact conformer(s), and SH3-m10 populates more highly charged, extended conformers to a far greater extent.

### Limited proteolysis followed by nESI-MS/MS analysis

**Figure 4:**
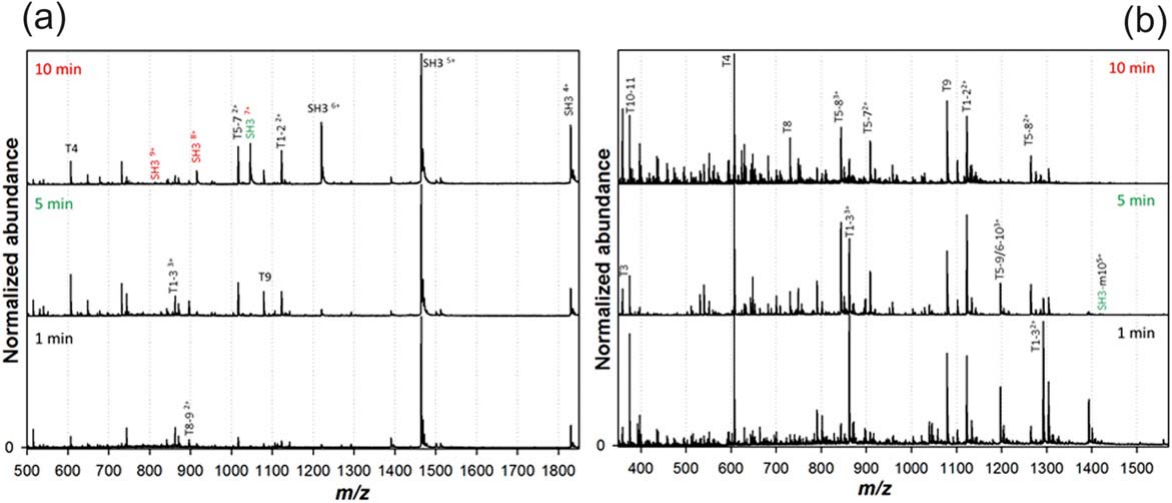
Trypsin proteolysis of SH3 and SH3-m10. Limited proteolysis (50 m*M* ammonium acetate pH 6.8, 20°C, protein:trypsin, 130 : 1, w/w) followed by ESI-MS analysis showing mass spectra of (a) SH3, and (b) SH3-m10, over 1, 5, and 10 min digest time.

To investigate how the structural differences between native SH3 and SH3-m10 affect their susceptibility to proteolysis, both proteins were subjected to trypsin digestion at pH 6.8 (protein:trypsin, 130:1, w/w). First, limited proteolysis followed by nESI-MS/MS analysis and sequencing of the resulting peptides was used to compare the conformational properties of the free proteins. Mass spectra of SH3 and SH3-m10 over 1 to 10 min digestion times under identical conditions (Fig. 4) indicate, as expected, that SH3 is more resistant to proteolysis than SH3-m10. No intact SH3-m10 was detected after 5 min digest time but, by contrast, the majority of the native SH3 remained intact even after 10 min of proteolysis.

To capture more specific structural details, the protein:trypsin ratios were optimized independently for both SH3 and SH3-m10 in order to identify the first trypsin cleavage site for each protein by time-resolved limited proteolysis (Supporting Information Fig. S3). For SH3 (protein:trypsin, 114:1 w/w), no peptides were detected until 3 min incubation, when the first peptide, covering the N-terminal residues 1–22 arising from cleavage at R22 (peptide labelled T1–3) was observed (Supporting Information Fig. S3a). The labelling nomenclature T1–3 indicates that this is the first tryptic (T) peptide starting from the N-terminus with cleavage occurring at the third lysine/arginine residue. To determine whether this observation could be related to the conformational properties of SH3, the solvent-accessible surface area (SASA) of each residue in SH3 (PDB 1SHG) was calculated (Supporting Information Fig. S4). After scaling to account for the variation in the sizes of different amino acid side-chains, R22 has the highest SASA of all residues in SH3. The detection of this peptide indicates that the first two basic residues (K7 and K19) remain uncut at this early time-point and hence are less susceptible to cleavage than R22. Observation of this peptide first is consistent with the location of R22 in the long interconnecting loop between the first two β-strands of SH3 26 being the region of the protein that is most susceptible to proteolysis, whilst the other potential cleavage sites (K7, K19, K27, K28, K40, K44, R50, K60 and K61) are less exposed to trypsin proteolysis. After 4 min cleavage, in addition to peptide T1–3, a small amount of peptide T4–9 (residues 23–60, following cleavage at both R22 and K60) was detected. This suggests that trypsin cleaves SH3 at R22 first, then the remaining C-terminal fragment peptide 23–63 is cleaved further at K60, based on the proposed proteolysis mechanism of folded proteins.^27^ After 5 min of digestion, further proteolytic products were identified as peptides T9 (residues 51–60, following cleavage at R50 and K60), T8–9 (residues 45–60, cleavage at K44 and K60), and T1–2 (residues 1–19, cleavage at K19), in addition to small amounts of peptide T5–7 (residues 29–44, cleavage at K28 and K44) [Fig. 4(a) and Supporting Information Fig. S3a]. After 10 min, additional peptides T4 (residues 23–27, cleavage at R22 and K27) and T6–10 (residues 29–61, cleavage at K28 and K61), were observed. By 20 mins, cleavage at K60 was also detected with the appearance of peptide T1–9 (residues 1–60). However, under these specific limited proteolysis conditions, trypsin digestion was not detected at either residue K7 or K40. Inspection of the SASA values (Supporting Information Fig. S4) indicates that K7 and K40 both have relatively high SASA scores in native SH3, so this does not account for the lack of proteolysis at these two residues. K7 is located immediately prior to the first β-strand of SH3 and K40 just before the third β-strand, so it is possible that these residues are protected by the secondary structure and/or dynamics of the protein in these regions.

By contrast with SH3, SH3-m10 is more susceptible to trypsin digestion, undergoing proteolysis at a much faster rate under identical conditions. At a ratio of SH3-m10:trypsin (130 : 1 w/w), six of the ten potential trypsin proteolysis sites were cleaved after only 1 min incubation: namely, residues K19, R22, K27, K28, R50, and K60, resulting in the detection of peptides T1–2 (residues 1–19), T1–3 (residues 1–22), T4 (residues 23–27), T5–8 (residues 28–50), T6–10 (residues 28–61), T9 (residues 51–60) and T10–11 (residues 60–63) [Fig. 4(b)]. However, at this early time-point, no cleavage at K7, K40 or K44 was observed. Repeating the digest under optimized milder conditions, with an SH3-m10:trypsin ratio of 2000:1 (w/w), revealed more details about the pathway of protein cleavage. The most susceptible cleavage site was identified as K60 after 5 min incubation (Supporting Information Fig. S3b). The next trypsin cleavage site under these conditions was R22, followed by K27, K19, R50, K44, and K61. Similar to SH3 proteolysis, no cleavage was observed at K7 or K40, under these specific limited proteolysis conditions, despite the lack of structure in SH3-m10.

The time-resolved limited proteolysis MS data suggest that not only do SH3 and SH3-m10 undergo trypsin cleavage at different rates, but also with a different order of cut-sites (Supporting Information Fig. S3). If the peptides detected during time-resolved limited proteolysis are the product of a single digest, these data imply that R22, located in the long interconnecting loop in native SH3, is the most exposed region, while K60, at the C-terminus, is the most exposed region in SH3-m10 that is in the case of the disordered SH3-m10, its first cleavage site is not limited to the long unstructured loop.

### Comparing SH3-RNC and SH3-m10 RNC with their free polypeptides

To examine whether the ribosome imposes any conformational constraints or interacts with exposed nascent SH3 domain sequences, the dynamics of the free and ribosome-tethered proteins were compared directly using limited proteolysis in conjunction with ESI-MS/MS. Thus, free SH3 was exposed to limited proteolysis under identical conditions in the absence of the ribosome, as well as in the form of an SH3-RNC complex to interrogate nascent SH3 chain folding and dynamics. The nascent chain represents only ca. 0.3 % of the total mass of the RNC which gives rise to technical challenges for MS analysis as strong background peaks originating from ribosome digestion complicate data interpretation. However, carefully controlled experiments combined with the sensitivity of MS for sequencing lowly populated peptides were able to provide a valuable tool to measure the conformational properties of isolated SH3 and SH3-m10, as well as their nascent chains tethered to the ribosome.

The SH3-RNC:trypsin ratio was optimized at 20°C with a reaction time of 10 min using a gradual increase in the ratio of trypsin:RNC-SH3. Limiting the amount of trypsin but maintaining a short digestion time was required to be able to distinguish unambiguously between the peptides originating from the nascent SH3 and the ribosome, without interrupting the integrity of the ribosome. The RNC:protease ratio depends on the properties of the nascent chains under investigation.^8^,^28^,^29^ For these experiments, the RNC:trypsin ratio was optimized at 700:1 (w/w). Once a sufficient intensity of peptides was detected by MS, an equivalent amount of free protein was subjected independently to proteolysis under identical conditions. nESI-MS/MS was used to establish the mass and sequence of all the tryptic peptides observed.

**Figure 5:**
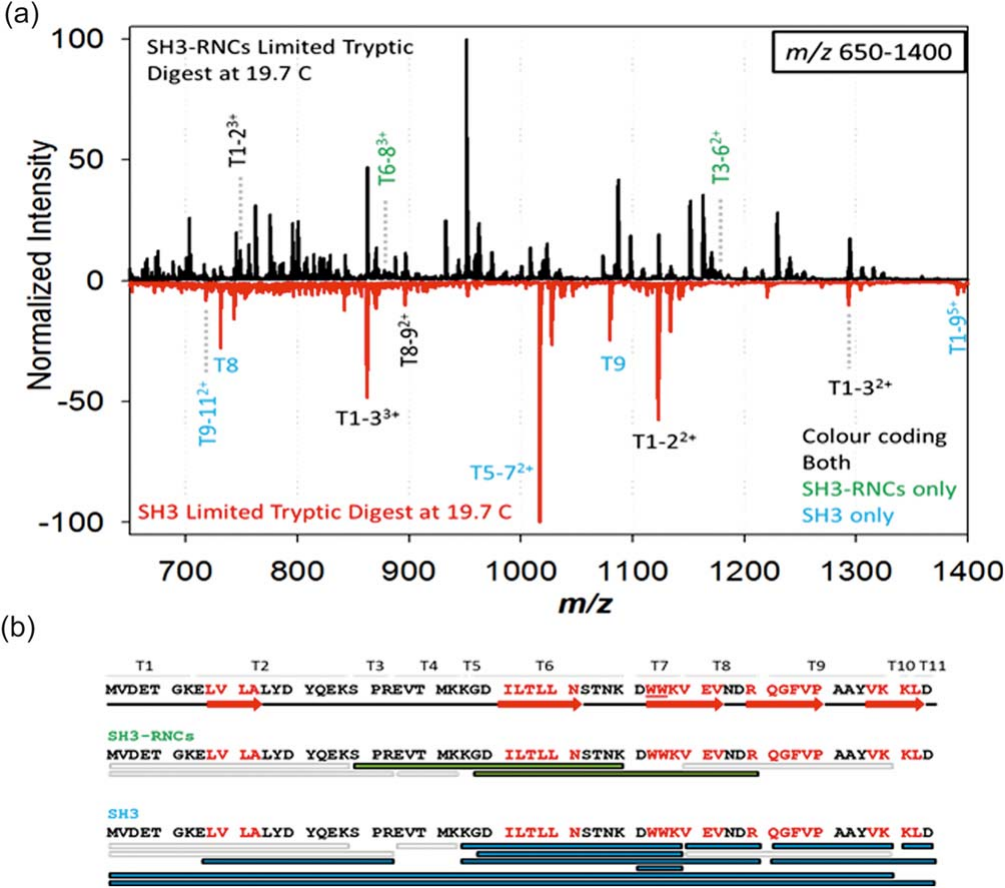
A comparison of limited proteolysis of SH3 and SH3-RNC. (a) ESI-MS spectra showing the peptide fragments detected following limited trypsin proteolysis (50 m*M* ammonium acetate, pH 6.8, 20°C) of SH3 (red peaks) and SH3-RNC (black peaks) (protein: trypsin, 700:1 w/w); (b) the amino acid sequence of SH3 showing the location of the five β-strands (red arrows) and the fragment maps of free SH3 and SH3-RNC. Peptides detected for SH3 only (blue); peptides detected for SH3-RNCs only (green); peptides detected for both proteins (gray).

A comparison of the data arising from the limited proteolysis of SH3-RNC and free SH3 in the range *m/z* 650 to 1400 is illustrated in Figure 5(a) and colour-coded with “black,” “blue,” and “green” representing peptides detected for both SH3 and SH3-RNC, SH3 only, and SH3-RNC only, respectively. It should be noted that the base peak and several of the major peaks in the mass spectrum of SH3-RNC originate from proteolysis products of the ribosome under these conditions. For both SH3 and SH3-RNC, peptides T1–2, T1–3, T4 and T8–9 were observed. Peptides T1–10, T2–3, T5–7, T5–8, T6–7, T7, T8, T9, T9–11, T10 and T1–9, were only observed in the spectrum of free SH3, whilst T3–6 and T6–8 were only detected in the spectrum of SH3-RNC. The tryptic fragment maps obtained by limited proteolysis of free SH3 and SH3-RNC are displayed in Figure 5(b) (gray bars indicate peptides detected for both SH3 and SH3-RNC; blue bars: peptides observed for SH3 only, and green bars: peptides observed for SH3-RNC only). The limited proteolysis data from free SH3 and SH3-RNC show some similarities between the peptide cleavage patterns at the N- and C-termini [Fig. 5(b)]. With the exception of the C-terminal residue K61 (which would cleave to generate a dipeptide that is likely too low in mass to be detected with confidence), all potential trypsin cleavage sites in free SH3 were identified; in the case of SH3-RNC, peptides originating from K7 cleavage (the first N-terminal cleavage site) were not detected, nor were peptides arising from K61 cleavage. The lack of trypsin cleavage at K7 indicates that the N-terminus of SH3 is more protected when the protein is an integral part of the RNC. Although, with the exception of K7, trypsin cleaves both free SH3 and SH3-RNC at similar sites, SH3 undergoes more extensive digestion throughout its sequence compared with SH3-RNC and the number of tryptic peptides detected for SH3-RNC is significantly lower than that observed for SH3 (Fig. 5). These findings are consistent with previous NMR studies which indicated that the SH3 domain tethered to the ribosome via SecM exhibits a native-like β-sheet structure.^7^

The N-terminus of SH3 exits the ribosome tunnel first and would be expected to remain close to the ribosome's outer surface after completion of synthesis and folding of the domain (as the N- and C-termini of SH3 are close in the native fold). One might postulate that the ribosome itself would provide a steric hindrance, shielding the nascent chain from trypsin. However, this postulation can be excluded as the sole reason for the different cleavage in the N-terminal region of SH3 compared with SH3-RNC as follows:

1. The SecM stall sequence with 27 amino acid residues spans one half of the ribosome tunnel^30^–^32^ (the tunnel can accommodate a maximum of 30 residues from the C-terminus of the nascent chain). The stalling peptide used in our RNCs has a total length of 27 amino acids,^7^ followed by an 8-residue linker, and a 14-residue length TEV cleavage site (Fig. 1). Altogether, this should ensure the exposure of nascent polypeptides well outside of the ribosome;
2. K60 is only three amino acids away from the C-terminus of SH3 and is cleaved in both SH3 and SH3-RNC. This indicates that the C-terminus of nascent SH3 is either exposed from the ribosome or the ribosome vestibule is accessible by trypsin (if the C-terminus resides in the vestibule). Considering a 9.51 Å distance between the C^α^ atoms of K7 and D63 in the crystal structure of the free SH3,^33^ the N-terminus should be in close proximity to the C-terminus once the SH3 domain folds when tethered to the ribosome.^7^

**Figure 6:**
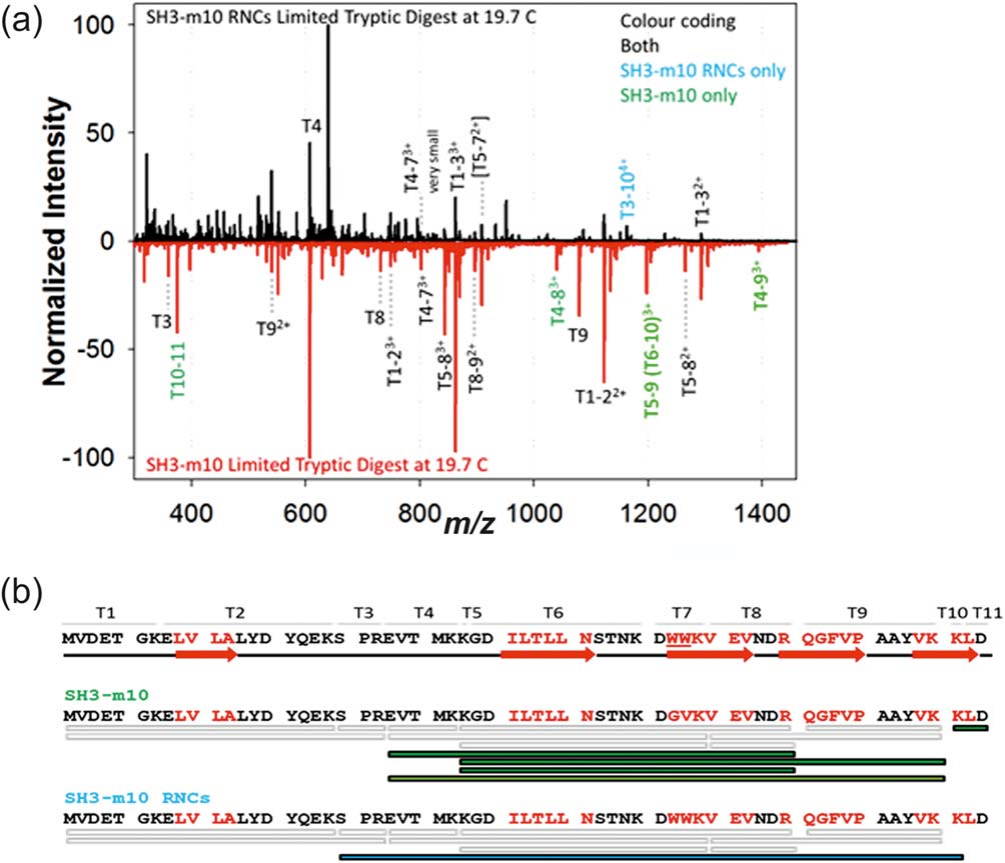
A comparison of limited proteolysis of SH3-m10 and SH3-m10 RNC. (a) ESI-MS spectra showing the peptide fragments detected following limited trypsin proteolysis (50 m*M* ammonium acetate, pH 6.8, 20°C) of SH3-m10 (red peaks) and SH3-m10 RNC (black peaks) (protein: trypsin, 700 : 1 w/w). (b) the amino acid sequence of SH3-m10 showing the location of the five β-strands in native SH3 (red arrows) and the fragment maps of free SH3-m10 and SH3-m10 RNC. Peptides detected for SH3-m10 only (green); peptides detected for SH3-RNCs only (blue); peptides detected for both (gray).

Next, the conformational properties of SH3-m10, as the isolated protein and a nascent chain complex, were compared using a similar limited proteolysis procedure. A higher degree of similarity was observed in the peptide fragmentation patterns of free and ribosome-bound SH3-m10 compared with the corresponding SH3 proteins. The mass spectra for both free SH3-m10 and SH3-m10-RNC show proteolysis products involving cleavage at seven out of the ten basic residues, namely: K19, R22, K27, K28, K44, R50 and K60 (Fig. 6). Further, peptides T4–8, T4–9, T5–8, T5–9 and T6–10 were detected solely in free SH3-m10 spectrum whereas peptide T3–10 was only observed in the SH3-m10-RNC spectrum. However, no peptides corresponding to K7 and K40 cleavages were detected for either free SH3-m10 or SH3-m10-RNC. This latter observation suggests that nascent SH3-m10 is exposed at its C-terminus, likely with no persistent interactions with the surface of the ribosome. Under identical limited proteolysis conditions, the majority of peptides detected in the limited proteolysis of free and ribosome-bound SH3-m10 are the same but of lower abundancy in the latter case. Comparing the limited proteolysis data obtained for nascent SH3-RNC with SH3-m10-RNC, the latter is more susceptible to trypsin cleavage, as would be expected for an unfolded ribosome-tethered chain.^7^

### Assessing the effect of the presence of RNCs on limited proteolysis of SH3 proteins

Finally, to determine whether the presence of the large number of lysine and arginine residues in the ribosomes themselves affects the trypsin cleavage patterns observed in SH3-RNC and SH3-m10-RNC, control experiments were performed in which RNCs containing a different nascent chain (GA98-RNCs^34^) were added to a solution of free SH3-m10 and the mixture subjected to limited proteolysis as before. The data show that the presence of GA98-RNC does not affect the tryptic digest products of free SH3-m10, as observed by nESI-MS/MS (Supporting Information Fig. S5).

## Conclusions

By using the SH3/SH3-m10 system, which has been studied previously at the atomic level using NMR^7^, as proof of principle we have demonstrated the utility of limited proteolysis coupled with MS to report on co-translational protein folding on the bacterial ribosome. The inherent high sensitivity, high speed of analysis and ability to analyze heterogeneous samples associated with MS allows short digestion times as only minute amounts of released peptides from the nascent chain are required for detection, thus minimizing time dependent changes in the nascent chain or ribosome conformation subsequent to proteolysis. The use of a specific protease such as trypsin enables straightforward identification of accessible locations on the nascent chain conformation, even in complex mixtures such as those described here. Differences in the resulting digestion fragments reveal small, but significant, variances in the conformational properties of the free proteins and permit comparisons to be made not only between wild-type and variant SH3, but also between the free proteins and their respective RNCs. These investigations pave the way for more detailed insights into nascent chain folding using chemical labelling techniques such as hydrogen-deuterium exchange, photochemical oxidation and cross-linking combined with MS analyses.
