# Supplementary material for: A comparison of the folding characteristics of free and ribosome-tethered polypeptide chains using limited proteolysis and mass spectrometry
Source: Protein Sci. 2015 Jun 11;24(8):1282–91. doi: 10.1002/pro.2702 (PMC4534179; doi:10.1002/pro.2702)

A comparison of the folding characteristics of free and ribosome-tethered polypeptide chains using limited proteolysis and mass spectrometry

Khadijeh Rajabi,[1] Julia Reuther,[2] Elke Deuerling,[2] Sheena E. Radford[1*], Alison E. Ashcroft.[1*]

[1]Astbury Centre for Structural Molecular Biology, School of Molecular and Cellular Biology, University of Leeds, Leeds LS2 9JT, United Kingdom.

[2] Department of Biology, University of Konstanz, 78457 Konstanz, Germany.

Supporting Information

Figure S1.

nESI-MS mass spectra of SH3 and SH3-m10 analysed from aqueous 10 mM ammonium acetate. SH3 (a) at pH 2.0 and (b) at pH 5.8; SH3-m10 (c) at pH 3.2 and (d) at pH 5.8. Charge state ions corresponding to +3 to +9 are indicated above the spectra.

n.b. The SH3-m10 used in these experiments was >90% pure by mass spectrometry. The small peaks m/z 1200-1400 are peptide impurities which were impossible to remove completely by standard purification procedures. These impurities (mass 3801 Da (+3 ions) and 5540 Da (+4 ions)) correspond to two partial sequences of SH3-m10: residues 21-54: PREVTMKKGDILTLLNSTNKDGVKVEVNDRQGFV and residues 6-54: GKELVLALYDYQEKSPREVTMKKGDILTLLNSTNKDGVKVEVNDRQGFV, respectively. As these minor impurities contain neither a C-terminal arginine or lysine, their presence does not affect the limited proteolysis results presented.

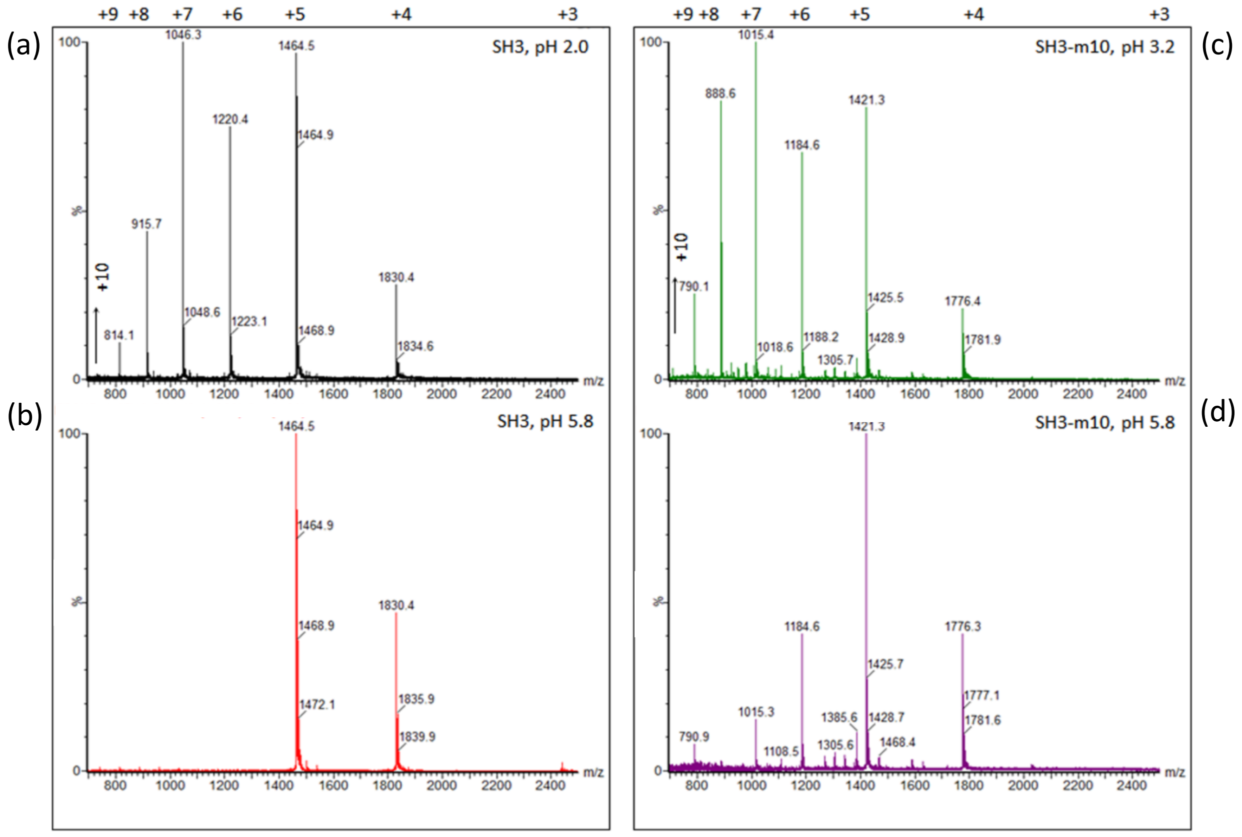

Figure S2.

nESI-IMS-MS collision cross-sectional (CCS) areas for SH3 (black crosses; charge states +4 to +6) and SH3-m10 (red circles; charge states +4 to +8). The theoretical CCS of native SH3 was calculated from PDB 1SHG as described previously (Smith D. P., Knapman T. W., Campuzano I, Malham R. W. Berryman, J. T., Radford, S. E., Ashcroft, A. E. (2009) Deciphering drift time measurements from travelling wave ion mobility spectrometry - mass spectrometry studies. Eur. J. Mass Spectrom. 15: 113-130). The difference between the calcuated CCS (750 Å^2) and the CCS of the most compact conformer detected for the lowest charge state (933 Å^2) is larger than the expected experimental error (± 7%; ± 65 Å^2) indicating some expansion of SH3 in the gas-phase.

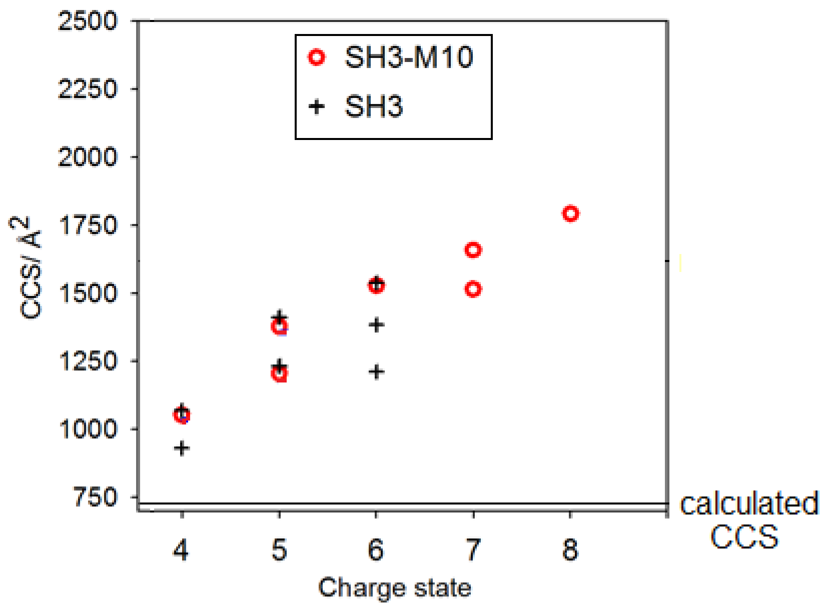

Figure S3.

(a). Limited trypsin proteolysis time-courses of (a) SH3 (protein:trypsin, 114:1 w/w) and (b) SH3-m10 (protein:trypsin, 2000:1 w/w), in 50 mM ammonium acetate, pH 6.8, 20 ^{o}C, as determined using nESI-MS/MS. The grey vertical bars show the times at which proteolysis at specific lysine and arginine residues are observed.

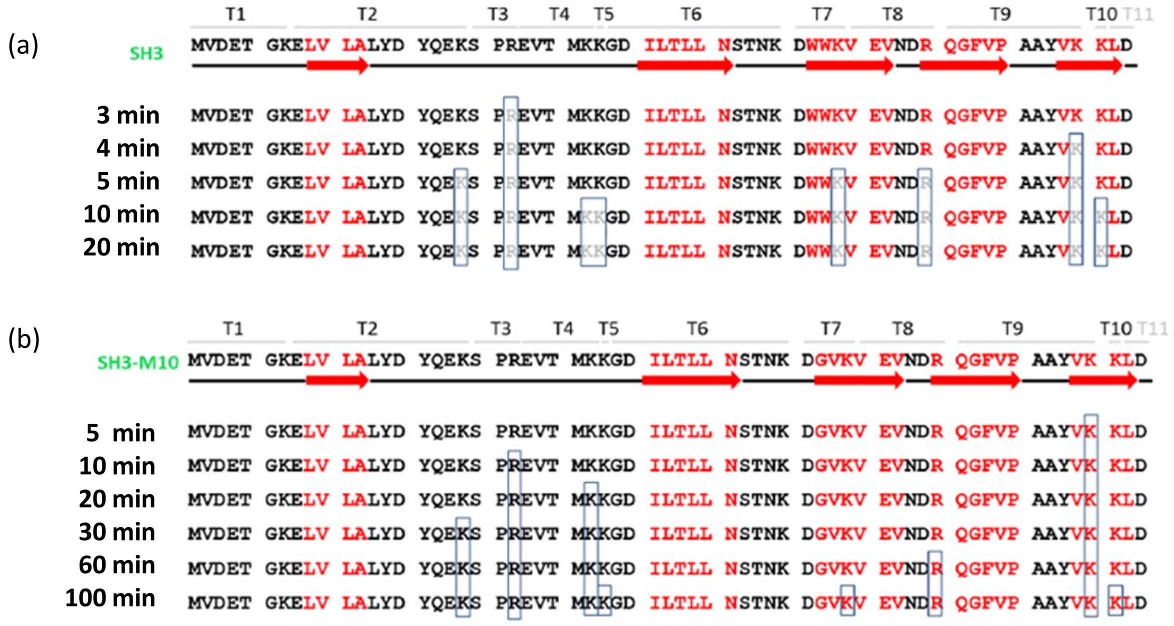

Figure S4.

Graph showing the solvent-accessible surface area (SASA; Å^2) for each residue of SH3 (PDB 1SHG). The individual residues were scaled to account for the variation in the sizes of each amino acid type. n.b. Recombinant SH3 has an extra N-terminal methionine residue (residue 1); residues 2 to 7 (VDETGK) are missing in the crystal structure of SH3 (PDB 1SHG). The blue asterisks (*) indicate the locations of the lysine and arginine residues in the SH3 sequence. The amino acid sequence for SH3 is shown below the graph, together with the locations of the five beta-strands observed in the native structure (red arrows and red font).

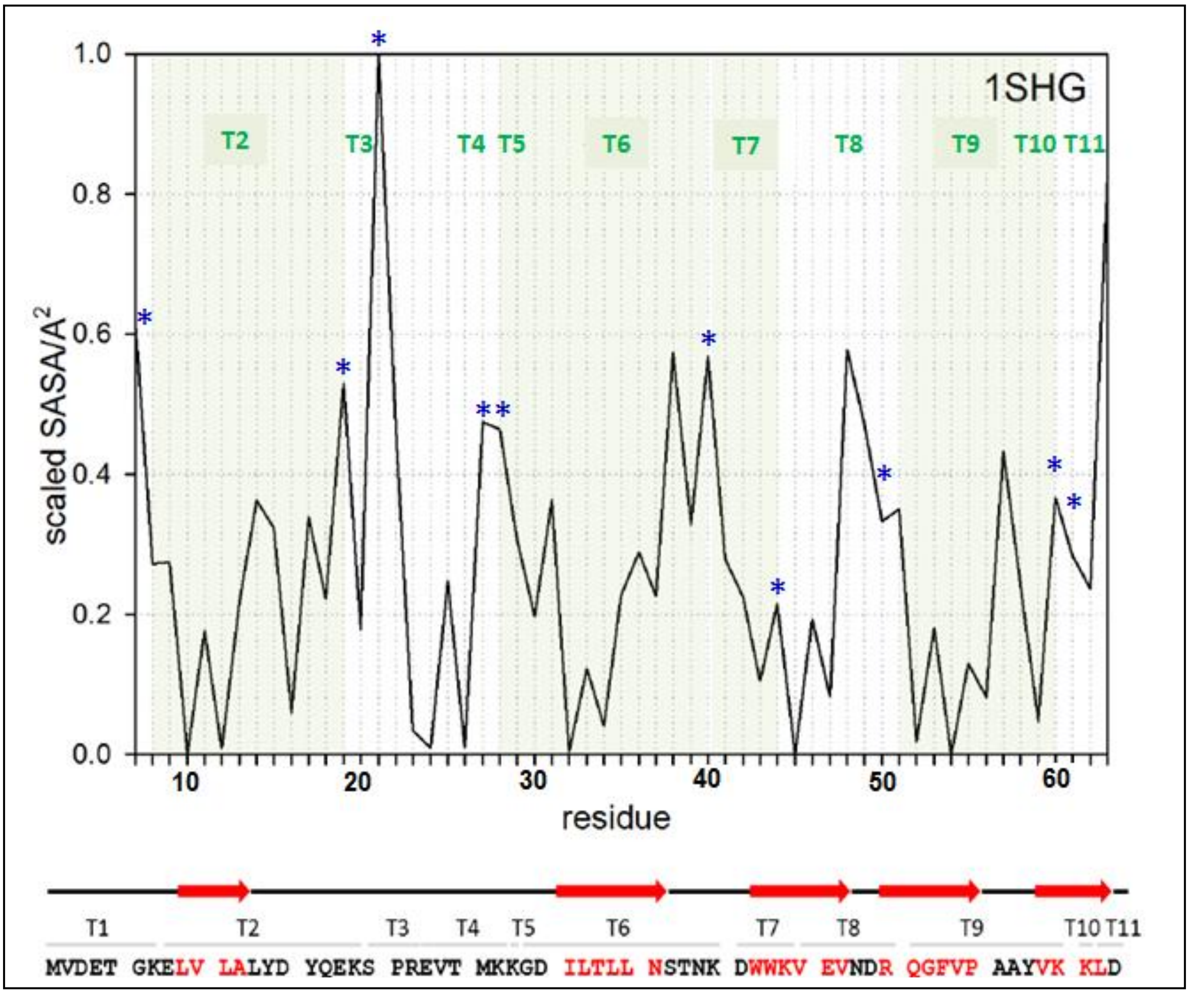

Figure S5.

nESI-MS spectra of the limited trypsin proteolysis (50 mM ammonium acetate, pH 6.8, 20 °C) observed for SH3-m10 (lower spectrum, red) and SH3-m10 in the presence of GA98 RNCs (upper spectrum, black font) (protein: trypsin 700:1 w/w). The similarities between the two spectra indicate that the presence of RNCs alone does not affect the proteolysis pattern of isolated SH3-m10.

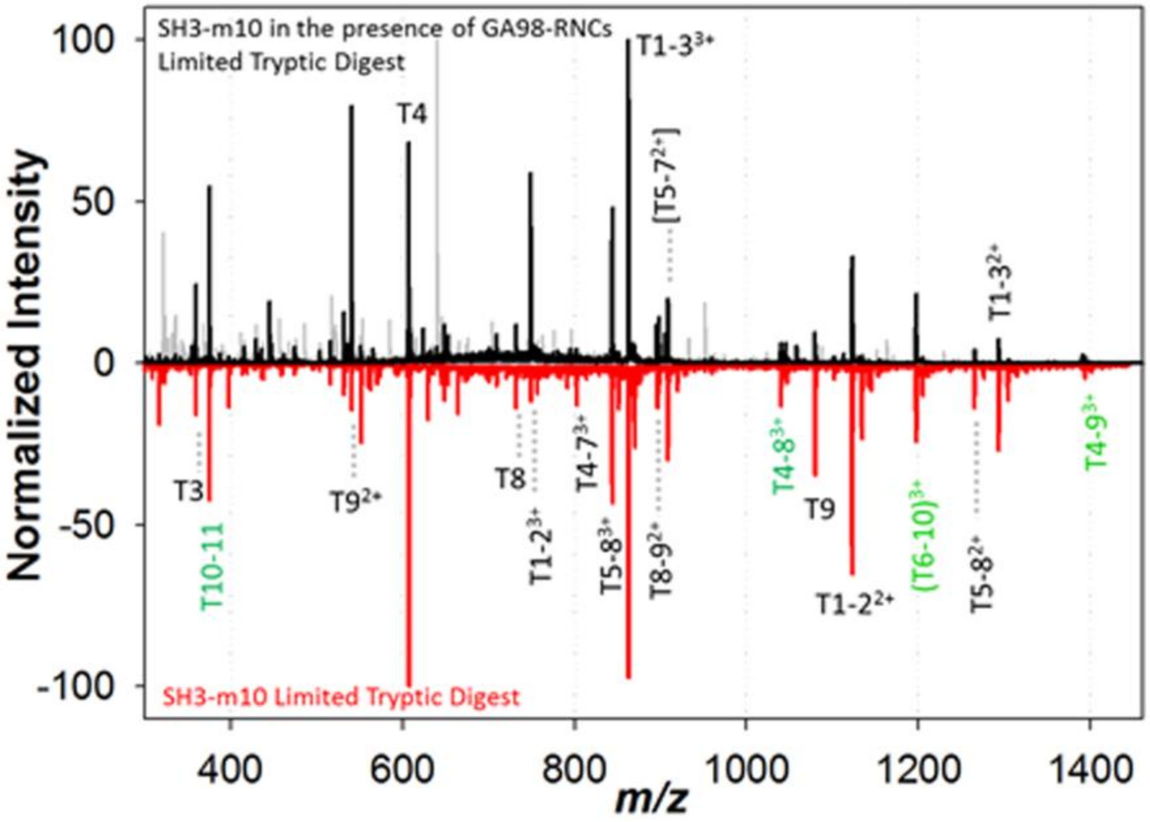

Supplement: Supplementary file 1 [file pro0024-1282-sd1.pdf]
